# Experiences of water immersion during childbirth: a qualitative thematic synthesis

**DOI:** 10.1186/s12884-023-05690-7

**Published:** 2023-05-29

**Authors:** E. Reviriego-Rodrigo, N. Ibargoyen-Roteta, S. Carreguí-Vilar, L. Mediavilla-Serrano, S. Uceira-Rey, S. Iglesias-Casás, A. Martín-Casado, A. Toledo-Chávarri, G. Ares-Mateos, S. Montero-Carcaboso, B. Castelló-Zamora, N. Burgos-Alonso, A. Moreno-Rodríguez, N. Hernández-Tejada, C. Koetsenruyter

**Affiliations:** 1 Osteba, Health Technology Assessment, Knowledge Management and Evaluation, Basque Foundation for Health Innovation and Research (BIOEF), Barakaldo-Bizkaia, Spain; 2 La Plana University Hospital, Villarreal-Castellón, Spain; 3 Osakidetza, OSI Debabarrena, Mendaro Hospital, Mendaro-Gipuzkoa, Spain; 4 Hospital da Barbanza, Ribeira-A Coruña, Spain; 5 Hospital do Salnés, Vilagarcía de Arousa-Pontevedra, Spain; 6 Universidad Internacional de La Rioja UNIR, Logroño-La Rioja, Spain; 7 Canary Islands Health Research Institute Foundation, Network for Research on Chronicity, Primary Care, and Health Promotion (RICAPPS), The Spanish Network of Agencies for Health Technology Assessment and Services of the National Health System (RedETS), Madrid, Spain; 8 Pediatrics Department, Hospital Universitario Rey Juan Carlos, Madrid, Spain; 9 Documentary of the Department of Health of the Basque Government, Territorial Delegation of Health of Bizkaia, Bilbao, Spain; 10 Department of Preventive Medicine and Public Health, Faculty of Medicine and Nursery, UPV/EHU, ES, Leioa, Spain; 11 Osakidetza, OSI Araba, Hospital Txagorritxu, Vitoria-Gasteiz, Spain; 12 People with Intellectual Disabilities, Vitoria-Gasteiz, Spain; 13 Mayan Center, Maternity, Yoga and Accompaniment, Bilbao, Spain

**Keywords:** Natural childbirth, Waterbirth, Water Immersion, Childbirth, Labor

## Abstract

**Background:**

The increasing demand for childbirth care based on physiological principles has led official bodies to encourage health centers to provide evidence-based care aimed at promoting women’s participation in informed decision-making and avoiding excessive medical intervention during childbirth. One of the goals is to reduce pain and find alternative measures to epidural anesthesia to enhance women’s autonomy and well-being during childbirth. Currently, water immersion is used as a non-pharmacological method for pain relief.

This review aimed to identify and synthesize evidence on women’s and midwives’ experiences, values, and preferences regarding water immersion during childbirth.

**Methods:**

A systematic review and thematic synthesis of qualitative evidence were conducted. Databases were searched and references were checked according to specific criteria. Studies that used qualitative data collection and analysis methods to examine the opinions of women or midwives in the hospital setting were included. Non-qualitative studies, mixed-methods studies that did not separately report qualitative results, and studies in languages other than English or Spanish were excluded. The Critical Appraisal Skills Program Qualitative Research Checklist was used to assess study quality, and results were synthesized using thematic synthesis.

**Results:**

Thirteen studies met the inclusion criteria and were included in this review. The qualitative studies yielded three key themes: 1) reasons identified by women and midwives for choosing a water birth, 2) benefits experienced in water births, and 3) barriers and facilitators of water immersion during childbirth.

**Conclusions:**

The evidence from qualitative studies indicates that women report benefits associated with water birth. From the perspective of midwives, ensuring safe water births requires adequate resources, midwives training, and rigorous standardized protocols to ensure that all pregnant women can safely opt for water immersion during childbirth with satisfactory results.

**Supplementary Information:**

The online version contains supplementary material available at 10.1186/s12884-023-05690-7.

## Background

Childbirth is a significant event in a woman’s life, with short- and long-term consequences that extend beyond her own health. It can also impact the well-being of her child and family, as well as her future reproductive choices and mode of delivery. A long-term follow-up study has found that positive birth experiences can enhance a woman’s self-confidence and self-esteem throughout her life [1].

In recent years, the demand for care based on the physiology of childbirth has prompted official bodies to encourage evidence-based care in health centers, aimed at empowering women to make informed decisions and minimizing obstetric intervention and medicalization during childbirth. One of the objectives is to reduce pain and explore alternative measures to epidural analgesia that increase women’s autonomy and well-being during childbirth. One such measure is water immersion, which is currently being used as a non-pharmacological method of pain relief [2].

The Cochrane systematic review “Immersion in water during labor and birth,“ by Cluett et al, defines “water immersion” as the practice of submerging a pregnant woman’s abdomen in water during any stage of labor, including dilation, expulsive, and delivery. On the other hand, “water birth” refers to the delivery of the newborn underwater [3].

Examining the experiences of mothers and midwives with water immersion is crucial, given the current emphasis on evidence-based care, efficient resource management, and the evaluation of a more humane model that reduces unnecessary interventions during labor. By reducing the need for medical interventions, water immersion may provide a more natural and positive birth experience for both mother and baby.

## Methods

The objective of this qualitative synthesis of evidence was to investigate the experiences of women and midwives with water immersion during labor.

### Systematic review of evidence

We conducted a systematic review of qualitative and mixed-methods studies, utilizing the SPIDER acronym (Sample, Phenomenon of Interest, Design, Evaluation, and Research type) to guide our review [4].

Our study sample included nulliparous or multiparous women in labor with singleton pregnancies who were healthy and at low risk of complications. In addition, we also included midwives and other professionals who were involved in obstetric care. The focus of our investigation was on the phenomenon of interest, which pertains to the experiences of women and midwives during water birth. Our study was limited to research conducted in hospital settings.

We considered published qualitative studies, studies with mixed methods designs, and surveys with free-text answer options, provided that the qualitative data could be extracted separately and had been formally analyzed using structured approaches such as thematic analysis or content analysis. We assessed the results by analyzing the narrative perspectives, experiences, and viewpoints of both pregnant women and midwives.

Our review included primary research studies and systematic reviews of qualitative studies published in English or Spanish. By synthesizing and analyzing these studies, we aimed to provide a comprehensive understanding of the experiences of women and midwives with water immersion during labor.

### Criteria for considering studies for this review

#### Inclusion criteria

We included studies that utilized qualitative research methods, such as ethnographic observations, in-depth interviews, focus group discussions, and open-ended survey questions. Studies with appropriate analysis methods, including thematic analysis, narrative analysis, framework analysis, and grounded theory, were also included [5]. Mixed-methods studies were only considered if they clearly described their qualitative data collection and analysis methods and provided in-depth findings and interpretations. We limited our review to studies published from 2009 to 2022. Including papers from 2009 allowed for a comprehensive review of literature on water immersion in labor and birth, as the first Cochrane review by Cluett et al. in that year was a significant milestone in the development of research in this area.

#### Exclusion criteria

We have excluded studies conducted outside the hospital setting, such as home births, from our analysis. Additionally, we have excluded studies that were published in languages other than English or Spanish.

By carefully selecting studies that met our inclusion criteria and excluding those that did not, we aimed to ensure that our review provided a comprehensive and high-quality synthesis of the experiences of women and midwives with water immersion during labor.

#### Search methods for identification of studies

We conducted a comprehensive search to identify all relevant studies, updating it until August 2022. We limited our search to studies published in English or Spanish from 2009 onwards. We searched several databases, including The Cochrane Library (Wiley), Centre for Reviews and Dissemination (CRD) [Database of Abstracts of Reviews of Effects (DARE)], Pubmed/Medline, Embase (OvidWeb), Web of Science (WOS), PsycINFO (OvidWeb), and Cinahl (EBSCOhost), using a combination of controlled and free language terms, such as “Labor”, “Natural Childbirth”, “Waterbirth” or “Water immersion”. The search strategies were adapted to each database, with the use of MESH descriptors and qualifiers to increase specificity when necessary. Alerts were set up in Medline (PubMed) and Embase (OVID) to identify any documents published up to August 2022. We also manually searched the literature cited in the selected studies to locate any relevant information not retrieved in the previous steps.

After completing the searches, we removed any duplicate citations, and the remaining records were uploaded to RefWorks reference manager. To assist with preparing systematic reviews, we used Ryyan, a software designed for this purpose.

#### Selection of studies

**Fig. 1:**
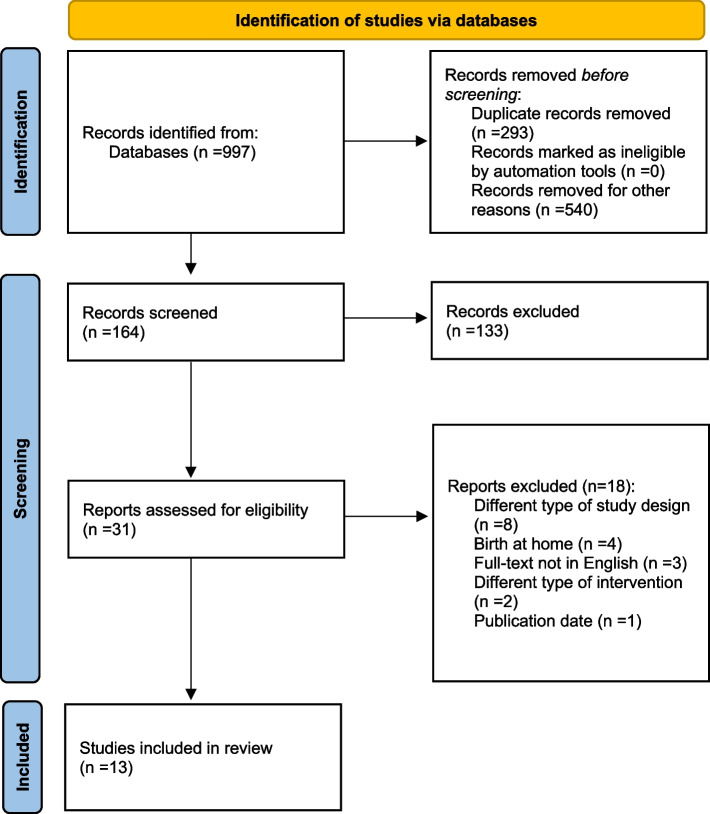
PRISMA flow chart of included studies

We imported all search results into Rayyan, and removed any duplicates. Subsequently, two review authors independently assessed the retrieved search results against the inclusion criteria. This screening process involved two stages: first, screening titles and abstracts, and then assessing the full-text articles. Employing two reviewers to screen the studies was advantageous as it allowed for an in-depth exploration of the relevance and meaning of the study findings. To arrive at a final selection, we held discussions until a consensus was reached, based on the study eligibility criteria. The entire screening process is summarized in a PRISMA flow diagram (Fig. 1), which outlines the number of studies removed and retained at each stage.

#### Quality appraisal/ assessment of methodological limitations

Prior to comparing findings and reaching a consensus, two reviewers conducted an assessment of methodological limitations for each paper using the Spanish version of the Critical Appraisal Skills Programme tool for qualitative studies (CASPe) [6]. Any disagreements between the reviewers were addressed and discussed until a consensus was reached. It is important to note that we did not exclude any studies based on our assessment of methodological limitations.

In the case of the systematic review of qualitative studies, the “Enhancing transparency in reporting the synthesis of qualitative research” tool [7] was applied.

#### Data extraction and thematic synthesis

We employed a standardized data collection form to extract the relevant data. Thematic synthesis was conducted, following the approach developed by Thomas and Harden [5]. To ensure a comprehensive analysis, all text in the results or findings sections of the included studies, including participant quotations and interpretations by the authors of the studies, were treated as data.

The lead reviewer (ER) extracted the data into tables and assigned codes to each line of text, based on its meaning and content, in accordance with the method outlined by Thomas and Harden [5]. These codes were then organized into descriptive themes, some of which corresponded with the original findings of the included studies. Next, the codes were grouped into logical and meaningful clusters in a hierarchical tree structure to form descriptive themes and sub-themes. Finally, the descriptive themes were developed into analytical themes, which enabled us to extend the analysis beyond the original studies.

#### Internal/external review

The project’s research team conducted an internal review of the work. After completing this stage, the work underwent an external review process, with recognized experts in the field providing feedback to ensure its quality, accuracy, and validity. Before participating in the review, the experts completed a document declaring any potential conflicts of interest.

## Results

### Included studies and quality assessment

Thirteen studies were identified for the review using PRISMA process (Fig. 1).

### Characteristics of the included studies

The 13 studies included in this analysis were published between 2013 and 2020, and 9 investigated both the first and second stages of labor, while 4 studies focused solely on the second stage of labor. Eight countries are represented across the studies, Australia (*n* = 4), United Kingdom (*n* = 2), Sweden (*n* = 2), Canada (*n* = 1), Portugal (*n* = 1), Greece (*n* = 1), Scotland (*n* = 1), and UU.EE (*n* = 1). Methodological approaches varied and qualitative methods used for purposes of data collection from women and midwives, most commonly involved interviews.

Nine studies focussed on women’s experience of water immersion during childbirth (Clews et al., 2019; Poder et al., 2020; Fair et al., 2020; Gonçalves et al., 2019; Lewis et al., 2018; Ulfsdottir et al., 2018; Antonakou et al., 2018; McKenna et al., 2013; Carlsson et al., 2020) [8–16], one study (Milosevic et al., 2019) [17] that explores the factors that determine the use of immersion during childbirth according to the point of view of both women and midwives and medical professionals (obstetricians, neonatologists and pediatricians), and three more studies on midwives’ experience with water immersion during childbirth (Cooper et al., 2019; Lewis et al., 2018; Nicholls et al., 2016 [18–20].

Tables 1, 2 and 3 provide a detailed overview of the study characteristics, including information about the author(s)/country, date, study design, participants, method of data collection, method of analysis, recruitment method and setting, study focus, and main findings.

**Table 1:** Summary of qualitative papers exploring women´s experiences of waterbirth

Author(s)/Country	Date	Design	Participants	Method of data collection	Method of analysis	Recruitment method and setting	Study focus	Findings
Clews et al. [8] United Kingdom	2019	A systematic meta-synthesis of qualitative studies	5 studies, which included a total of 346 women	In-depth Interviews Semi-structured Interviews Skype audio semi-structured interviews Interviews Questionnaire Interviews	Meta-ethnography, reciprocal translational análisis	Hospitals, researchers and online social media	To explore women’s experiences of waterbirth	Four themes were identified: women’s knowledge of waterbirth; women’s perception of physiological birth; water, autonomy and control; and waterbirth: easing the transition.
Poder et al. [9] Canada	2020	A mixed-method approach, combining systematic reviews of the literature and patient focus groups to identify attributes and levels explaining women’s preferences.	17 women	Focus groups Questionnaire	Discrete choice experiment	Convenience sampling method, recruited through the research team’s personal and professional networks	To identify the factors that are most significant for women when deciding whether to give birth in water or not	The study considered six attributes: birth mode, duration of the labor phase, pain sensation, risk of severe tears in the perineum during the expulsion of the newborn, risk of death of the newborn, and general condition of the newborn (Apgar) score at 5 min.
Fair et al. [10] UU.EE.	2020	Qualitative	23 women	Semi-structured interviews.	Grounded theory	The women were recruited from an obstetrics and gynecology center in a mid-sized city in the south eastern United States.	To investigate the decision-making process of women who choose to give birth in water	Three main themes emerged: Beliefs and desires; actions and agency; empowerment and accomplishment
Gonçalves et al. [11] Portugal	2019	Qualitative, phenomenological study	13 women	Semi-structured interviews	Content analysis	Online purposive selection to recruit participants for this study	To gain a deeper understanding of the experiences of mothers who have undergone one or multiple water births	The following categories were presented: water benefits; and manifestations of satisfaction with the experience of physiological labour in water by the woman.
Lewis et al. [12] Australia	2018	Qualitative exploratory design	296 women	Telephone interviews.	Thematic analysis	Publicly-funded hospital located in Western Australia	To explore the perceptions and experiences of women who have given birth, regardless of their decision to opt for a water birth or not	The benefits of planning a water birth are numerous, including pain relief, a desire for a natural birth, the potential for a calming and peaceful environment, and recommendations from healthcare professionals. However, the most significant factor in ensuring a successful water birth is having a supportive team present. Midwives are often cited as the primary source of support, which is essential for the mother’s well-being and the successful delivery of the baby. Participants described their water birth experiences using a range of adjectives, such as empowering, unforgettable, quick, natural, firming, painful, and lengthy.
Ulfsdottir et al. [13] Sweden	2018	Qualitative	20 women	In-depth interviews	Content analysis	From a clinic located in Stockholm	To investigate and describe the perceptions and experiences of women who have given birth while immersed in water	Three categories were found: “Synergy between body and mind”, “Privacy and discretion”, and “Natural and pleasant”.
Antonakou et al. [14] Greece	2018	Qualitative	12 women	Individual interviews	Thematic analysis	An open invitation through the website birthscientist.gr, a scientific society that promotes natural childbirth in Greece	To explore the essential concepts and themes that arise from analyzing the experiences of women who underwent labor and delivery while immersed in water	Three main themes were identified: Water use as a natural way of birth; mixed messages from the healthcare professionals, and; partner’s supportive role during water birth.
McKenna et al. [15] Scotland	2013	Qualitative	8 women	Semi-structured interviews	Interpretative phenomenological analysis	From the obstetric unit of a Scottish healthcare center	To investigate the utilization of water immersion as a delivery method for women who have had a previous cesarean section and desire to have a vaginal birth in water	The study revealed three prominent themes: “minimizing,“ “maximizing,“ and “managing.“ The participants’ interviews uncovered the following themes: Firstly, the women reported that water birth helped minimize medical intervention during childbirth. Secondly, they emphasized that water birth maximized their experience in four crucial ways: physical benefits, psychological benefits, choice, and control. Finally, the third theme related to how women managed the potential risks associated with water birth, along with navigating the expectations and behaviors of their families, friends, and healthcare professionals involved in their care.
Carlsson et al. [16] Sweden	2020	Qualitative	111 women	Online questionnaire interviews with open-ended questions	Qualitative content analysis.	From two hospitals in Sweden.	To explore retrospective descriptions about benefits, negative experiences and information related to waterbirths	Two themes were identified related to benefits: (a) physical benefits: the water eases labour progression while offering buoyancy and pain relief; and (b) psychological benefits: improved relaxation and control in a demedicalized and safe setting. Two themes were identified related to negative experiences: (a) equipment-related issues due to the construction of the tub and issues related to being immersed in water; and (b) fears and worries related to waterbirth. In regard to preparatory information, respondents reported a lack of general and specific information related to waterbirths, even after they contacted birthing units to ask questions.

**Table 2:** Summary of qualitative papers exploring women´s and midwives´s experiences of waterbirth

Author(s)/Country	Date	Design	Participants	Method of data collection	Method of analysis	Recruitment method and setting	Study focus	Findings
Milosevic et al. [17] United Kingdom	2019	Qualitative descriptive	**85 women** 83.5% used a pool or bath in labor 63.3% had given birth in water **21 midwives** 11 clinical midwives 5 midwifery managers 5 consultant midwives/clinical specialists **14 medical staff** 7 consultant obstetricians 1 trainee obstetrician 5 consultant neonatologists 1 consultant paediatrician	Online discussion groups (women and midwives) Semi-structured interviews (medical staff)	Thematic analysis	The recruitment method for this study was opportunistic, with calls for participation disseminated through various online platforms	To identify the factors that influence the use of wáter birth during childbirth	Factors influencing the use of birth pools were grouped into three overarching categories: re- sources, unit culture and guidelines, and staffendorsement. Resources encompassed pool availability, effi- ciency of pool use and availability of waterproof cardiotocograph equipment. Unit culture and guidelines related to eligibility criteria for pool use, medicalisation of birth and differences between midwifery-led and obstetric-led care. Staffendorsement encompassed attitudes towards pool use.

**Table 3:** Summary of qualitative papers exploring midwives´s experiences of waterbirth

Author(s)/Country	Date	Design	Participants	Method of data collection	Method of analysis	Recruitment method and setting	Study focus	Findings
Cooper et al. [18] Australia	2019	A three-phase mixed methods approach.	12 policy and guideline informants 200 midwives	Documentary análisis Interviews Surveys	Convergent or parallel approach	Phase one data collection provided the majority of recruitment opportunities whilst referral and word-of-mouth provided further opportunities for recruitment. The survey was deployed through the Australian College of Midwives	To explore the development of policies and guidelines concerning water immersion during labor and birth, and analyze the experiences of policy and guideline informants in creating such policies and guidelines. Additionally, to gain insight into the perspectives and experiences of Australian midwives regarding water immersion, as well as their involvement in the development of policies and guidelines	Fur main themes arising from the examination of the participants’ experiences in developing and implementing WI policies and guidelines: (1) the burden of ‘proof’, (2) risk driven policies and guidelines to ensure safety, (3) autonomous control or controlling autonomy and (4) talking from experience.
Lewis et al. [19] Australia	2018	Mixed method study	12 midwives.	Questionnaire and two focus groups	Thematic analysis	This study was conducted at a birth centre of a tertiary public maternity hospital in Western Australia	To present an overview of the education, knowledge, and practices related to water immersion during labor or delivery among midwives in Western Australia, based on their experiences	Exploration of what midwives enjoyed about caring for women who used water immersion revealed three themes: instinctive birthing; woman-centred atmosphere; and undisturbed space. Exploration of the challenges experienced with waterbirth revealed two themes: learning through reflection and facilities required to support waterbirth.
Nicholls et al. [20] Australia	2016	Qualitative descriptive	26 midwives	Interviews and focus group	A modified grounded theory methodology with thematic analysis	The study participants were midwives from four different public hospitals	To capture midwives’ perceptions of becoming and being confident in conducting waterbirth in addition to factors perceived to inhibit and facilitate the development of that confidence	Three main categories emerged from the data analysis: what came before the journey, becoming confident – the journey and staying confident. Each contained between threea nd five subcategories.Together they depicted how midwives describe the journey to becoming confident to support women who have chosen the option to waterbirth and how they are able to retain that confidence once achieved.

Experiences of water immersion during childbirth: a qualitative thematic synthesis

The quality of these studies was evaluated using the CASPe tool, which is widely recognized as a reliable assessment method. For easy reference, the Supplementary Material includes summary tables that provide an overview of the quality of evidence presented in the included studies.

### Mothers’ experiences with water immersion during labor and birth

Clews et al. [8] conducted a metasynthesis of qualitative studies on women’s experiences with water birth. They found four primary themes, which included the mother’s knowledge of water birth, their perception of a physiologic birth, water, autonomy, and control, and water birth easing the transition. The authors concluded that water birth can be an empowering experience for those who choose it and reinforces women’s sense of autonomy and control during the birthing process.

The study conducted by Poder et al. [9] aimed to identify factors that influence women’s decision to choose water birth or not. They used focus groups to create a validated questionnaire utilizing Discrete Choice Experiments (DCE). The questionnaire considered various attributes that women consider important in making their decision, including type of delivery, duration of labor, pain sensation, risk of severe tearing, risk of newborn death, and general condition (Apgar score at 5 min).

The study by Fair et al. [10] explored the decision-making process of women who planned to give birth in water. Women sought information from the internet and social networks and desired to limit medical interventions during childbirth. Support from doulas and midwives played a critical role in their decision-making process, while many experienced resistance from family, friends, and colleagues. Although not all women gave birth in water, most reported positive experiences and felt empowered. They encouraged other women to consider water birth and expressed a desire to have a water birth in the future.

In the study conducted by Gonçalves et al. [11] in Portugal, semi-structured interviews were conducted with mothers who had experienced one or more water births before they were no longer offered by the public health system. The analysis resulted in the identification of seven categories, but the study primarily focuses on two categories: the benefits of water immersion during childbirth, including pain relief and the ability to witness the birth of the child, and the satisfaction of women with the experience.

The study conducted by Lewis et al. in 2018 [12] aimed to explore the motivations, facilitating and hindering factors, and the birth experiences of women who gave birth in water in a tertiary public hospital in Australia. Telephone interviews were conducted with 296 women 6 weeks after giving birth. Of the participants, only 31% were able to have a water birth, with multiparous women having a higher success rate than primiparous women. Women who planned for a water birth cited pain relief, preference, association with natural childbirth, calming atmosphere, and recommendation as reasons. Support, particularly from midwives, played a crucial role in the success of water birth. The study did not specify which obstetric complications prevented of the women from giving birth in water.

Ulfsdottir et al. [13] conducted in-depth interviews with primiparous and multiparous mothers three to five months after giving birth. and found that water birth created a comfortable, home-like space that helped women feel relaxed, safe, and in control during childbirth. Three categories emerged: “synergy between body and mind,“ “privacy and discretion,“ and “natural and pleasant.“ The study suggested that water birth could enhance the childbirth experience, but the hospital where the study was conducted provided ongoing support, which may have contributed to positive experiences regardless of whether participants had a water birth or not.

Antonakau et al. [14] conducted a study on the experiences of women who gave birth using water birth in private facilities in Greece. The study identified three themes: water birth is a natural way of giving birth, healthcare professionals give contradictory messages regarding water births, and the supportive role of partners during the process. All participants reported a positive experience, with water immersion helping them manage pain and feel empowered after birth, resulting in successful breastfeeding for over a year. However, women had difficulty finding healthcare professionals who supported their choices, while they felt very supported by their partners. It is important to note that the participants were a homogenous group, primarily older, more educated, and financially able to afford private maternity care.

McKenna’s study [15] explored the experiences of women who had a water birth after a previous cesarean section in a Scottish midwifery-led unit. The study found that water birth minimized medical intervention, maximized physical and psychological benefits, and allowed women to have greater control and choice during childbirth. The study also highlighted the women’s management of potential risks associated with water birth and their interactions with healthcare providers, family, and friends.

Carlsson et al. [16] study included women who gave birth in water. The study identified physical and psychological benefits, including pain relief and improved relaxation, as well as negative experiences such as equipment problems and concerns related to water birth. Participants noted a lack of reliable information on water births and had to seek supplementary information online. The study highlights the need for accessible and reliable information on water births.

### Mothers’ and midwives’ experiences with water immersion during labor and birth

Milosevic et al. [17] conducted a study to investigate factors that influence the use of water immersion during childbirth. The study employed online focus groups with women and midwives, as well as interviews with medical professionals. Eligibility criteria were found to limit access to water births, and obstetrician-led units were described as overly medicalized settings with limited provision of water births. Midwives were found to increase access to water births by proactively offering it as an option during childbirth and providing information to women about water birth during antenatal care.

### Midwives’ experiences with water immersion during labor and birth

Cooper, Nicholls, and Lewis have conducted three studies to investigate the experiences of midwives with water immersion during labor or birth [18–20]. These studies shed light on the benefits and challenges associated with water immersion, as well as the attitudes of midwives towards this birthing option. By examining midwives’ perspectives, these studies provide valuable insights into the implementation and promotion of water immersion.

A study by Cooper et al. [18] investigated the policies and guidelines for water immersion during labor and birth in Australia, as well as midwives’ experiences and perspectives. The study included a literature search, interviews, and an online questionnaire, and found that midwives must be accredited to facilitate water immersion to promote access to this option. However, midwives faced barriers related to accreditation and inconsistent guidelines across facilities. The study suggests the need for standardized guidelines and improved training opportunities for midwives to ensure the safe and effective use of water immersion during labor and birth. Overall, the study highlights the importance of promoting access to water immersion as a birthing option while ensuring appropriate training and guidelines for healthcare professionals.

Lewis et al. [19] examined midwives’ perceptions of their education, knowledge, and practice of water immersion during labor and birth in Australia. The study used a two-phase mixed-methods approach, including a questionnaire and focus groups. The results of the questionnaire showed that 93% of midwives felt confident attending water births after attending an average of seven water births, and they enjoyed facilitating water immersion. The focus groups identified several positive aspects of caring for women during water immersion, such as instinctive birth and a woman-centered environment, as well as challenges related to learning through observation and the need for support to enable water births. Overall, the study highlights midwives’ positive experiences and the importance of training and support to ensure safe and effective water immersion during labor and birth.

The study by Nicholls et al. [20] emphasizes the importance of midwives’ competence and confidence in supporting water births according to local clinical practice guidelines. Interviews with 16 midwives and a focus group with 10 others identified three categories related to confidence acquisition: pre-pathway factors, pathway to confidence, and maintenance of confidence. The study identified three categories that affect midwives’ confidence in supporting water births: 1) factors before entering the profession, 2) factors that contribute to confidence development, and 3) factors that help maintain confidence.

The study’s findings have three significant implications for midwifery practice. Firstly, it is recommended that graduate students and midwives work in maternity wards led by midwives who support normal physiological birth. Secondly, it is suggested that learning directly from experienced midwives who can address their specific needs would benefit maternity wards. Lastly, it is emphasized that midwives have a crucial role as “protectors” of normal physiological birth, and mandatory attendance at sessions highlighting this role and the current evidence supporting normal birth, including water immersion during labor, is necessary.

### Thematic synthesis

To summarize the most significant findings from the qualitative studies on water immersion in childbirth, several tables have been created based on the themes that emerged from the studies. Thematic synthesis identified the following three themes:

- *Theme 1. Reasons for choosing water birth.*
  This theme investigates the factors that influenced women’s decision to use water immersion during childbirth, such as pain relief, relaxation, and a desire for a more natural birth experience. Some professionals also cited benefits to the baby, such as reducing stress and facilitating a smoother transition to the outside world.
- *Theme 2. Benefits of water immersion.*
  This theme includes the positive experiences reported by women who used water immersion during labor and birth, such as reduced pain, increased relaxation, and a greater sense of control. Midwives and other health professionals also noted benefits, including improved maternal-fetal bonding and a decreased need for medical interventions.
- *Theme 3. Barriers and facilitators of water immersion:*
  This theme includes factors that can either hinder or promote the use of water immersion during childbirth. For example, midwives’ attitudes and training were identified as critical facilitators, while hospital policies and protocols were seen as significant barriers. Other factors included access to appropriate facilities and equipment, communication and coordination among healthcare providers, and support from partners and family members.

Tables 4, 5 and 6 provide a comprehensive overview of the themes and subthemes present in the included studies. These tables serve as a visual representation of the key concepts and ideas that emerged from the analysis of the data.

Table 4 presents the reasons for choosing a water birth based on the findings of the included studies.

**Table 4:** Reasons for choosing a water birth

Reasons identified	Source
Prior knowledge of water birth	[8, 12]
Recommendation by others	[8, 12]
Relaxation and decreased anxiety	[9, 12]
Sense of comfort and well-being	[9]
Desire for natural birth	[10, 12]
Pain relief during labor	[9, 12]
Reduced likelihood of perineal tearing	[9]
Shortened active phase of labor	[9]
No increased risk of newborn mortality compared to conventional delivery	[9]
No adverse effect on newborn’s general condition (Apgar test)	[9]
No increased risk of infection for the newborn	[9]

[8] Clews et al. 2019; [9] Poder et al. 2020; [10] Fair et al. 2020; [12] Lewis et al. 2018

The table includes 5 identified reasons for choosing water birth and the sources of the evidence supporting each reason. The reasons were identified by four studies: Clews et al. (2019), Poder et al. (2020), Fair et al. (2020), and Lewis et al. (2018) [8–10, 12].

The most common reasons for choosing a water birth, as reported the studies, include prior knowledge of water birth [8, 12], recommendation by others [8, 12], relaxation and decreased anxiety [9, 12], sense of comfort and well-being [9], desire for natural birth [10, 12], pain relief during labor [9, 12]. Other reported benefits of water birth include reduced likelihood of perineal tearing [9], shortened active phase of labor [9], no increased risk of newborn mortality compared to conventional delivery [9], no adverse effect on newborn’s general condition (Apgar test) [9], and no increased risk of infection for the newborn [9].

Overall, the table suggests that women may choose water birth for various reasons, such as personal preference and potential benefits, without increasing the risk of adverse outcomes for the newborn.

Table 5 summarizes the identified benefits of water birth, along with the sources of evidence supporting each benefit.

**Table 5:** Benefits of water birth identified by women/midwives

Benefits	Source
A greater feeling of autonomy and control over the childbirth process	[8, 11, 12, 15, 16, 19]
Increased opportunities for experiencing a more natural childbirth	[8, 10–14, 16, 19];
Easing the transition into motherhood	[8]
Providing pain relief without relying on medical interventions	[11, 12, 16]
Allowing the opportunity to witness the birth of the child	[11]
The option of immersion in water	[11, 12]
Immersion in water for increased mobility and a sense of lightness	[11, 16]
Tranquility, improved breathing, and relaxation leading to synergy between body and mind	[11–13, 16, 19];
More privacy and discretion during the childbirth process	[13]
Minimizing the medicalization of childbirth, resulting in less use of analgesia and oxytocin	[10, 12, 15–17]
A feeling of positive experience	[9, 10, 12, 16]
A feeling of success in childbirth	[11]
More physical and psychological benefits	[15, 16]

[8] Clews et al. 2019; [10] Fair et al. 2020; [11] Gonçalves et al. 2019; [12] Lewis et al. 2018; [13] Ulfsdottir et al. 2018; [14] Antonakou et al. 2018; [15] McKenna et al. 2013; [16] Carlsson et al. 2020; [17] Milosevic et al. 2019; [19] Lewis et al. 2018 (midwives)

Table 5 summarizes the reported benefits of water birth as identified by women who had experienced it, as well as midwives who have supported such births. The benefits include a greater feeling of autonomy and control over the childbirth process [8, 11–13, 16, 19], increased opportunities for experiencing a more natural childbirth [8, 10–14, 16, 19], easing the transition into motherhood [8], providing pain relief without relying on medical interventions [11, 12, 16], allowing the opportunity to witness the birth of the child [11], the option of immersion in water [11, 12], immersion in water for increased mobility and a sense of lightness [11, 16], tranquility, improved breathing, and relaxation leading to synergy between body and mind [11–13, 16, 19], more privacy and discretion during the childbirth process [13], minimizing the medicalization of childbirth, resulting in less use of analgesia and oxytocin [10, 12, 15–17], a feeling of positive experience [8, 10–12], a feeling of success in childbirth [11], and more physical and psychological benefits [15, 16].

Overall, the table highlights the various benefits of water birth as reported by women and midwives, including physical and psychological advantages, which may encourage women to consider water birth as a viable option for childbirth.

Additionally, Table 6 presents a comprehensive overview of the barriers and facilitators that have been identified in the studies analyzed.

**Table 6:** Barriers and facilitators to water immersion during childbirth

**Barriers to water immersion during childbirth**	*Source*
***Safety concerns***	
Potential risks associated with waterbirth after cesarean section	[15]
Obstetric complications	[12]
***Cultural factors***	
Lack of support from family members and health professionals	[10]
***Limited resources***	
Lack of necessary equipment or facilities to support waterbirth	[12]
**Facilitators of water immersion in childbirth**	
*** Resources***	
Availability of bathtubs and appropriate usage techniques	[17]
Availability of cardiotocographic equipment compatible with water immersion	[17]
*** A culture of support for water immersion during childbirth***	
Clear and consistent eligibility criteria for water immersion during childbirth	[17]
Promoting a natural childbirth experience without unnecessary medical interventions	[15, 17]
*** Support for the mother***	
Support from health professionals	[10, 12, 14, 17, 20]
Support for partners during water immersion childbirth, including emotional and physical support	[14]
*** Training and support for healthcare professionals who attend water immersion childbirths, including proper techniques and safety precautions***	
Learning through observation of successful water immersion childbirths to improve technique and confidence	[12]
Professional waterbirth training and senior staff support	[17, 18, 20]
Positive impact of coworker presence during water immersion childbirths on patient outcomes and healthcare provider satisfaction	[20]
Confidence to improve with the experience of attending such births	[18, 20]
Provision of clear and accurate information to pregnant women about the benefits and risks of water immersion childbirths	
Promotion of water immersion through education	[16, 17]

[10] Fair et al. 2020; [12] Lewis et al. 2018; [14] Antonakou et al. 2018; [15] McKenna et al. 2013; [16] Carlsson et al. 2020; [17] Milosevic et al. 2019; [18] Cooper et al. 2019; [18] Lewis et al. 2018 (midwives); [20] Nicholls et al. 2016

Table 6 provides a summary of the barriers and facilitators identified in the studies included in this analysis related to water immersion during childbirth. The references in the table include several studies that investigated water immersion during childbirth, such as Fair et al. 2020 [10], Lewis et al. 2018 [12], Antonakou et al. 2018 [14], McKenna et al. 2013 [15], Carlsson et al. 2020 [16], Milosevic et al. 2019 [17], Cooper et al. 2019 [18], Lewis et al. 2018 (midwives) [19], and Nicholls et al. 2016 [20].

The table lists various factors that could either impede or promote water immersion during childbirth.

The barriers to water immersion during childbirth identified in this study include safety concerns related to potential risks associated with waterbirth after cesarean section [15] and obstetric complications [12]. The lack of support from family members and healthcare professionals was also identified as a barrier to water immersion during childbirth [10].

On the other hand, several facilitators of water immersion in childbirth were identified, including the availability of bathtubs and appropriate usage techniques [17], clear and consistent eligibility criteria for water immersion during childbirth [17], and support from health professionals [10, 12, 14, 17, 20]. Moreover, training and support for healthcare professionals attending water immersion childbirths, including proper techniques and safety precautions, also facilitate successful water immersion childbirths [12, 17, 18, 20].

Finally, provision of clear and accurate information to pregnant women about the benefits and risks of water immersion childbirths, promotion of water immersion through education [16, 17], and a culture of support for water immersion during childbirth were identified as crucial facilitators for successful water immersion childbirths.

To ensure safe water births, midwives emphasize the importance of having adequate resources, consistent protocols, specialized training, and a supportive culture towards water immersion during childbirth. This support should come from all healthcare professionals involved in the birth process, not just midwives. By ensuring these factors are in place, midwives can confidently attend water births and provide the best care for the mother and baby.

## Discussion

This article examines qualitative studies that explore the experiences of women and healthcare teams caring for mother-newborn pairs.

The results of the qualitative studies suggest that women who choose to use water immersion during labor often have a positive and empowering experience, leading to a more natural childbirth and increased satisfaction [8]. However, the studies also identified various barriers such as potential obstetric complications, lack of support from family members and healthcare professionals, and inadequate resources and facilities for water births. To offer this service safely and effectively, facilities must have proper equipment, maintenance and cleaning protocols, action protocols, and contingency plans for potential complications. Healthcare professionals must also receive specialized training in water birth practices, and the resource must be readily available upon request [10, 12].

One of the studies [9] examined the factors that can influence a woman’s decision to have a water birth and found that the most significant factors were pain reduction, the risk of neonatal mortality, the risk of severe perineal tears, slightly better general condition of the newborn (as indicated by the Apgar test), and reduction of the duration of the active phase of labor. The study also highlighted the importance of providing accurate and comprehensive information to pregnant women about water immersion during childbirth, as many women reported not receiving enough information on this option.

Two studies conducted by Carlsson et al. (2020) [16] and Milosevic et al. (2019) [17] revealed the insufficient provision of information about water births during antepartum classes and midwife consultations. Furthermore, it is essential to incorporate the systematic collection of data obtained from the use of water birth to address the quality-of-care indicators during childbirth. This approach will ensure that pregnant women who choose water births during labor receive the highest level of safe and quality care.

During the final stage of writing this article, the study by Feeley C. et al., 2021 [21] was retrieved through alerts. This study is a meta-synthesis of qualitative studies and used GRADE-CERQual [22] to evaluate the results. The meta-synthesis included seven studies to evaluate the impact of water immersion during labor [11, 13–15, 23–25], out of which four were part of the systematic review [11, 13–15]. The findings revealed that women who used water immersion during any stage of labor facilitated women’s physical and psychological needs, offering effective analgesia and a versatile tool that women can adapt and influence to best suit their individual needs. Women who used warm water immersion for labor and/or birth described the experience as liberating, transformative, and empowering, resulting in a positive birth experience. Based on these results, the study suggests that maternity professionals and services should improve women’s access to water immersion and offer it as a standard method to of pain relief during labor for low-risk pregnant women.

## Conclusions

- Qualitative studies have consistently shown that women who have experienced water births associate numerous benefits with the practice. These benefits include reduced pain and discomfort during labor, a greater sense of relaxation and control, increased satisfaction with the birth experience, and improved maternal and fetal outcomes. Additionally, water immersion during labor has been found to reduce the need for pharmacological pain relief, interventions such as episiotomy, and operative deliveries. These findings highlight the potential benefits of water immersion as a safe and effective option for women during labor and delivery.
- Midwives emphasize the importance of adequate resources, standardized and rigorous protocols, training for midwives, and a supportive culture for water immersion during childbirth, with input from all professionals involved in attending the birth, including those who care for both mothers and newborns.
- It is recommended to improve the information provided to women regarding pain relief options, establish common protocols for water births in NHS hospitals, standardize training for these deliveries, and increase human and material resources to ensure that all pregnant women have the possibility of safely and satisfactorily using hot water immersion during labor, regardless of their location.

## Supplementary Information

**Additional file 1.**

## Data Availability

All data generated or analysed during this study are included in this published article [and its supplementary information files].
