# Supplementary material for: Experiences of water immersion during childbirth: a qualitative thematic synthesis
Source: BMC Pregnancy Childbirth. 2023 May 29;23:395. doi: 10.1186/s12884-023-05690-7 (PMC10226235; doi:10.1186/s12884-023-05690-7)

**Experiences of water immersion during childbirth: a qualitative thematic synthesis**

Supplementary Material’

I. Search strategies

**Limit - since 2009**

**Sources:**

**The Cochrane Library (Wiley)**

**The Cochrane Pregnancy and Childbirth Group's Trials Register**

**Cochrane Central Register of Controlled Trials (CENTRAL)**

#1 MeSH descriptor: MeSH descriptor: [Natural Childbirth] explode all trees

#2 (waterbirth*):ti,ab,kw (Word variations have been searched)

#3 #1 OR #2

**Documents retrieved=5**

Search terms: waterbirth*

**Limit– since 2009**

**Centre for Reviews and Dissemination (CRD) [Database of Abstracts of Reviews of Effects (DARE)]**

**Documents retrieved=2**

Search terms: waterbirth*

**Limit– since 2009**

**Clinical Trials- Clinical Trial Registry (U.S. National Institutes of Health)**

**Documents retrieved=1**

**Pubmed/Medline**

**1 Search** ("Natural Childbirth/adverse effects"[Mesh] OR "Natural Childbirth/epidemiology"[Mesh] OR "Natural Childbirth/methods"[Mesh] OR "Natural Childbirth/nursing"[Mesh] OR "Natural Childbirth/psychology"[Mesh] OR "Natural Childbirth/statistics and numerical data"[Mesh])

**2** **Search:** ((("Water"[Mesh]) OR "Immersion"[Mesh]) OR "Baths"[Mesh]) OR "Hydrotherapy"[Mesh]

**3 Search: #1 AND #2**

**4 Search:** ( Clinical Trial Protocol[ptyp] OR Guideline[ptyp] OR Meta-Analysis[ptyp] OR Practice Guideline[ptyp] OR Randomized Controlled Trial[ptyp] OR Review[ptyp] OR systematic[sb] )

**5 Search: #3 AND #4**

**Search synthesis**

((("Natural Childbirth/adverse effects"[Mesh] OR "Natural Childbirth/epidemiology"[Mesh] OR "Natural Childbirth/methods"[Mesh] OR "Natural Childbirth/nursing"[Mesh] OR "Natural Childbirth/psychology"[Mesh] OR "Natural Childbirth/statistics and numerical data"[Mesh] ))) AND (((("Water"[Mesh]) OR "Immersion"[Mesh]) OR "Baths"[Mesh]) OR "Hydrotherapy"[Mesh]) AND ( ( Clinical Trial Protocol[ptyp] OR Guideline[ptyp] OR Meta-Analysis[ptyp] OR Practice Guideline[ptyp] OR Randomized Controlled Trial[ptyp] OR Review[ptyp] OR systematic[sb]) "

**6 Search**: (("Labor, Obstetric"[Mesh]) OR "Analgesia, Obstetrical"[Mesh]) OR "Delivery, Obstetric"[Mesh]) OR "Midwifery"[Mesh]))

**7 Search: #6 AND #2 AND #4**

**Search synthesis**

(("Labor, Obstetric"[Mesh] OR "Analgesia, Obstetrical"[Mesh] OR "Delivery, Obstetric"[Mesh] OR "Midwifery"[Mesh]) AND ((("Water"[Mesh] OR "Immersion"[Mesh]) OR "Baths"[Mesh]) OR "Hydrotherapy"[Mesh])) AND (Clinical Trial Protocol[ptyp] OR Guideline[ptyp] OR Meta-Analysis[ptyp] OR Practice Guideline[ptyp] OR Randomized Controlled Trial[ptyp] OR Review[ptyp] OR systematic[sb]) AND ("2009/07/20"[PDat])

**8 Search**: (((("Qualitative Research"[Mesh]) OR "Empirical Research"[Mesh]) OR "Health Services Research"[Mesh]) OR "Systematic Reviews as Topic"[Mesh])

**9 Search**: **#3 AND #8**

**Search synthesis**

(((((((("Water"[Mesh]) OR "Immersion"[Mesh]) OR "Baths"[Mesh]) OR "Hydrotherapy"[Mesh])) AND (( "Natural Childbirth/adverse effects"[Mesh] OR "Natural Childbirth/epidemiology"[Mesh] OR "Natural Childbirth/methods"[Mesh] OR "Natural Childbirth/nursing"[Mesh] OR "Natural Childbirth/psychology"[Mesh] OR "Natural Childbirth/statistics and numerical data"[Mesh] ))) AND (((((("Qualitative Research"[Mesh]) OR "Empirical Research"[Mesh]) OR "Health Services Research"[Mesh]) OR "Systematic Reviews as Topic"[Mesh]))

**10 Search: #2 AND #6 AND #8**

**Search synthesis**

((((((("Water"[Mesh]) OR "Immersion"[Mesh]) OR "Baths"[Mesh]) OR "Hydrotherapy"[Mesh]) AND (((("Labor, Obstetric"[Mesh]) OR "Analgesia, Obstetrical"[Mesh]) OR "Delivery, Obstetric"[Mesh]) OR "Midwifery"[Mesh])) AND (((((("Qualitative Research"[Mesh]) OR "Empirical Research"[Mesh]) OR "Health Services Research"[Mesh]) OR "Systematic Reviews as Topic"[Mesh]))

**11 Search**:  (bath OR bathtub OR hydrotherapy OR water OR immersion OR birthing pool*). **Field**: Title/Abstrac]

**12 Search**: labour OR labor OR parturition OR Childbirth. **Field:** Title/Abstract

**13 Search**: **#11 AND #12**

**14 Search: #13 AND #4**

**Search synthesis**

((((((((bath[Title/Abstract]) OR bathub[Title/Abstract]) OR hydrotherapy[Title/Abstract]) OR water[Title/Abstract]) OR immersion[Title/Abstract]) AND (((((labour[Title/Abstract]) OR labor[Title/Abstract]) OR parturition[Title/Abstract]) OR Childbirth[Title/Abstract]) AND (( Clinical Trial Protocol[ptyp] OR Guideline[ptyp] OR Meta-Analysis[ptyp] OR Practice Guideline[ptyp] OR Randomized Controlled Trial[ptyp] OR Review[ptyp] OR systematic[sb] OR Qualitative ))

**15 Search: # 13 AND #8**

**Search synthesis**

((((((((bath[Title/Abstract]) OR bathub[Title/Abstract]) OR hydrotherapy[Title/Abstract]) OR water[Title/Abstract]) OR immersion[Title/Abstract]) AND (((((labour[Title/Abstract]) OR labor[Title/Abstract]) OR parturition[Title/Abstract]) OR Childbirth[Title/Abstract]) AND ((((("Qualitative Research"[Mesh]) OR "Empirical Research"[Mesh]) OR "Health Services Research"[Mesh]) OR "Systematic Reviews as Topic"[Mesh]))

**16 Search**: "Water Birth" OR Waterbirth OR "Birth in water" OR "underwater birth" OR "giving birth in water" OR "Water immersion labour" OR "labouring in water " OR “Immersion in water during labor”

**17 Search: (#4 OR #8) AND #16**

**Search synthesis**

((("Water Birth" OR Waterbirth OR "Birth in water" OR "underwater birth" OR "giving birth in water" OR "Water immersion labour" OR "labouring in water " OR “Immersion in water during labor”) AND ((((( Clinical Trial Protocol[ptyp] OR Guideline[ptyp] OR Meta-Analysis[ptyp] OR Practice Guideline[ptyp] OR Randomized Controlled Trial[ptyp] OR Review[ptyp] OR systematic[sb] ))) OR ((((("Qualitative Research"[Mesh]) OR "Empirical Research"[Mesh]) OR "Health Services Research"[Mesh]) OR "Systematic Reviews as Topic"[Mesh])))

**18 Search:** Qualitative OR analysis OR interview OR questionnaire OR survey OR focus groups OR observation OR record OR ethnography OR phenomenological OR case study OR field study OR hermeneutic OR mixed methods study OR exploratory design OR critical incident techniques OR experiences OR expectations OR perceptions OR preferences OR perspectives OR opinions OR attitude OR grounded theory

**19 Search: #16 AND # 18 AND #4**

**Search synthesis**

 ((((("Water Birth" OR Waterbirth OR "Birth in water" OR "underwater birth" OR "giving birth in water" OR "Water immersion labour" OR "labouring in water " OR “Immersion in water during labor”)) AND ((qualitative OR analysis OR interview OR questionnaire OR survey OR focus groups OR observation OR record OR ethnography OR phenomenological OR case study OR field study OR hermeneutic OR mixed methods study OR exploratory design OR critical incident techniques OR experiences OR expectations OR perceptions OR preferences OR perspectives OR opinions OR attitude OR grounded theory) AND (( Clinical Trial Protocol[ptyp] OR Guideline[ptyp] OR Meta-Analysis[ptyp] OR Practice Guideline[ptyp] OR Randomized Controlled Trial[ptyp] OR Review[ptyp] OR systematic[sb] )) Sort by: Best Match Filters: Clinical Trial; Clinical Trial Protocol; Guideline; Meta-Analysis; Practice Guideline; Randomized Controlled Trial; Review; Systematic Reviews

**20 Search:** Immersion Water During Labor Delivery

**21 Search #20 AND # 4**

**Documents retrieved=635**

**Embase, vía OvidWeb**

**1 Search:** **basic search**: waterbirth {Including related terms}. ("qualitative (maximizes sensitivity)" and yr="2009 -Current")

**2 Search**: Advanced search: waterbirth.mp. or exp water birth/. limit to (embase status and yr="2009 -Current") ("reviews (maximizes sensitivity)" or "reviews (maximizes specificity)" or "reviews (best balance of sensitivity and specificity)" or "qualitative (maximizes sensitivity)" or "qualitative (maximizes specificity)" or "qualitative (best balance of sensitivity and specificity)")

**3 Search** : **fields**: waterbirth.ab,hw,kw,ot,sh,ti.

**Documents retrieved=55**

**Psycinfo, vía OvidWeb**

**1 Search**: waterbirth.ab. or waterbirth.id. or waterbirth.ti

**2 Search**: waterbirth {Including Related Terms}

Filter = 16 references.

**3 Search**: Multi-field search

"Water Birth" OR Waterbirth OR "Birth in water" OR "underwater birth" OR "giving birth in water" OR "Water immersion labour" OR "labouring in water " OR “Immersion in water during labor”

**Documents retrieved=31**

**Cinahl, vía EBSCOhost**

**"**water birth OR waterbirth OR underwater birth OR giving birth in water OR Water immersion labour OR labouring in water OR Immersion in water during labor; Consultas clínicas: Review - High Sensitivity, Review - High Specificity, Review - Best Balance, Qualitative - High Sensitivity, Qualitative - High Specificity, Qualitative - Best Balance

**Documents retrieved=150 references**

**WOS**

1 Search: TEMA: ("water birth") OR TEMA: (waterbirth) OR TEMA: ("underwater birth") OR TEMA: ("giving birth in water") OR TEMA: ("Water immersion labour") OR TEMA: ("labouring in water") OR TEMA: ("Immersion in water during")

Bases de datos= WOS, CCC, KJD, SCIELO Limit=2009-2022=

2 Searh TEMA: ("water birth") OR TEMA: (waterbirth) OR TEMA: ("underwater birth") OR TEMA: ("giving birth in water") OR TEMA: ("Water immersion labour") OR TEMA: ("labouring in water") OR TEMA: ("Immersion in water during")

**Documents retrieved=30 references**

II. Quality assessment of included studies.

Tabla S1. CASPe Qualitative Studies Checklist: experiences of women.

|  | **Poder et alet al. 2020** | **Fair et al. 2020** | **Gonçalves et al. 2019** | **Lewis et al. 2018** | **Ulfsdottir et al. 2018** | **Antonakou et al. 2018** | **McKenna et al. 2013** | **Carlsson 2020** |
| --- | --- | --- | --- | --- | --- | --- | --- | --- |
| 1. Was there a clear statement of the aims of the research | Yes | Yes | Yes | Yes | Yes | Yes | Yes | Yes |
| 2. Is a qualitative methodology appropriate? | Yes | Yes | Yes | Yes | Yes | Yes | Yes | Yes |
| 3. Was the research design appropriate to address the aims of the research? | Yes | Yes | Yes | Yes | Yes | Yes | Yes | Yes |
| 4. Was the recruitment strategy appropriate to the aims of the research? | Yes | Yes | Yes | Yes | Yes | Yes | Yes | Yes |
| 5. Was the data collected in a way that addressed the research issue? | Yes | Yes | Yes | Yes | Yes | Yes | Yes | Yes |
| 6. Has the relationship between researcher and participants been adequately considered? | Can’t Tell | Yes | Can’t Tell | Can’t Tell | Can’t Tell | Can’t Tell | Can’t Tell | Can’t Tell |
| 7. Have ethical issues been taken into consideration? | Yes | Yes | Yes | Yes | Yes | Yes | Yes | Yes |
| 8. Was the data analysis sufficiently rigorous? | Yes | Yes | Yes | Yes | Yes | Yes | Yes | Yes |
| 9. Is there a clear statement of findings? | Yes | Yes | Yes | Yes | Yes | Yes | Yes | Yes |
| 10. How valuable is the research? | Yes | Yes | Yes | Yes | Yes | Yes | Yes | Yes |

All studies met the criteria of the CASPE tool except for the reflexivity question, which was not fully addressed in any of the studies during the assessment.

Table S2. CASPe Qualitative Studies Checklist: experiences of women and midwives.

|  | **Milosevic et al. 2019** |
| --- | --- |
| 1. Was there a clear statement of the aims of the research | Yes |
| 2. Is a qualitative methodology appropriate? | Yes |
| 3. Was the research design appropriate to address the aims of the research? | Yes |
| 4. Was the recruitment strategy appropriate to the aims of the research? | Yes |
| 5. Was the data collected in a way that addressed the research issue? | Yes |
| 6. Has the relationship between researcher and participants been adequately considered? | Can’t Tell |
| 7. Have ethical issues been taken into consideration? | Yes |
| 8. Was the data analysis sufficiently rigorous? | Yes |
| 9. Is there a clear statement of findings? | Yes |
| 10. How valuable is the research? | Yes |

During the assessment, this study was found to meet the criteria of the CASPE tool, with the exception of the reflexivity question, which was not adequately addressed.Table S3. CASPe Qualitative Studies Checklist: experiences of widwives.

|  | **Cooper et al. 2019** | **Lewis et al. 2018** | **Nicholls et al. 2016** |
| --- | --- | --- | --- |
| 1. Was there a clear statement of the aims of the research | Yes | Yes | Yes |
| 2. Is a qualitative methodology appropriate? | Yes | Yes | Yes |
| 3. Was the research design appropriate to address the aims of the research? | Yes | Yes | Yes |
| 4. Was the recruitment strategy appropriate to the aims of the research? | Yes | Yes | Yes |
| 5. Was the data collected in a way that addressed the research issue? | Yes | Yes | Yes |
| 6. Has the relationship between researcher and participants been adequately considered? | Can’t Tell | Can’t Tell | Can’t Tell |
| 7. Have ethical issues been taken into consideration? | Yes | Yes | Yes |
| 8. Was the data analysis sufficiently rigorous? | Yes | Yes | Yes |
| 9. Is there a clear statement of findings? | Yes | Yes | Yes |
| 10. How valuable is the research? | Yes | Yes | Yes |

All studies met the criteria of the CASPE tool except for the reflexivity question, which was not fully addressed in any of the studies during the assessment.

Table S4. SR of qualitative studies.

According to an evaluation using the "[Enhancing Transparency in Reporting the Synthesis of Qualitative Research](https://www.sbu.se/contentassets/14570b8112c5464cbb2c256c11674025/methodological_limitations_qualitative_evidence_synthesis.pdf)" tool, the study conducted by Clews et al. in 2019 (8) satisfied 73% of the criteria (8 out of 11).


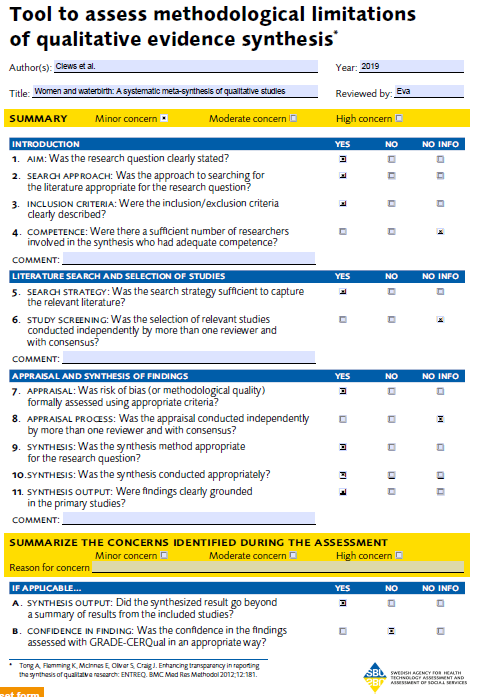

Supplement: Supplementary file 1 — Additional file 1. [file 12884_2023_5690_MOESM1_ESM.docx]
